# A single-center, open-label trial on convenience and complications of rechargeable implantable pulse generators for spinal cord stimulation: The Recharge Pain Trial

**DOI:** 10.1007/s10143-022-01940-y

**Published:** 2023-01-14

**Authors:** Mohammad Mehdi Hajiabadi, Petya Vicheva, Andreas Unterberg, Rezvan Ahmadi, Martin Jakobs

**Affiliations:** 1 Department of Neurosurgery, University Hospital Heidelberg, Heidelberg, Germany; 2 Division of Operative Pain Management, Department of Neurosurgery, University Hospital Heidelberg, Heidelberg, Germany; 3 Division of Stereotactic Neurosurgery, Department of Neurosurgery, University Hospital Heidelberg, Im Neuenheimer Feld 400, D-69120 Heidelberg, Germany

**Keywords:** Spinal cord stimulation, Implantable pulse generators, Rechargeable pulse generators, Questionnaire-based, Pain therapy

## Abstract

Rechargeable implantable pulse generators (r-IPGs) have been available for spinal cord stimulation (SCS) claiming to offer a longer service life but demanding continuous monitoring and regular recharging by the patients. The aim of the study (DRKS00021281; Apr 7^th^, 2020) was to assess the convenience, safety, and acceptance of r-IPGs and their effect on patient lives under long-term therapy. Standardized questionnaires were sent to all chronic pain patients with a r-IPG at the time of trial. Primary endpoint was the overall convenience of the charging process on an ordinal scale from “very hard” (1 point) to “very easy” (5 points). Secondary endpoints were charge burden (min/week), rates of user confidence and complications (failed recharges, interruptions of therapy). Endpoints were analyzed for several subgroups. Data sets *n* = 40 (42% return rate) were eligible for analysis. Patient age was 57.2 ± 12.6 (mean ± standard deviation) years with the r-IPG being implanted for 52.1 ± 32.6 months. The overall convenience of recharging was evaluated as “easy” (4 points). The charge burden was 112.7 ± 139 min/week. 92% of the patients felt confident recharging the neurostimulator. 37.5% of patients reported failed recharges. 28.9% of patients experienced unintended interruptions of stimulation. Subgroup analysis only showed a significant impact on overall convenience for different models of stimulators (*p* < 0.05). Overall, SCS patients feel confident handling a r-IPG at high rates of convenience and acceptable effort despite high rates of usage-related complications. Further technical improvements for r-IPGs are needed.

**Supplementary Information:**

The online version contains supplementary material available at 10.1007/s10143-022-01940-y.

## Introduction

Spinal cord stimulation (SCS) is an established neuromodulatory therapy for managing chronic and neuropathic trunk and extremity pain. SCS systems usually consist of a pulse generator as a power source providing the stimulation, one or multiple leads located in the spinal epidural space that transmit the stimulation onto the dorsal columns of the spinal cord and optionally an extension wire, which connects the lead to the PG [1, 2]. Since the inception of this technology more than 50 years ago, the devices have been markedly developed starting with the development of fully implantable pulse generators (IPG) powered by non-rechargeable primary cell batteries [3, 4]. While non-rechargeable IPGs (nr-IPG) offer relatively effortless therapy, their implant size is rather large and they require surgical IPG replacements every 2–5 years once the battery capacity has been depleted [1, 4]. The success and long-term therapy of SCS had led to the development to rechargeable IPGs (r-IPGs) and their introduction in 2004 [1, 4]. The use of r-IPGs has reduced the morbidity and potential costs associated with repeat surgical procedures [1]. To maintain continuous therapy, patients are required to regularly check the battery capacity of the r-IPG and perform a non-invasive recharging process. Failure to recharge the r-IPG will result in a complete drain of the battery and an unintended interruption of stimulation. The charging process is similar for all manufacturers of r-IPGs and consists of the following steps: (1) checking the residual capacity of the r-IPG with a handheld controller, (2) putting on a belt or harness that holds the charger which is placed on the skin above the r-IPG, (3) coupling the charger to the r-IPG to start the charging process, (4) keeping the connection between the charger and the r-IPG during inductive charging, and (5) charging the charger at a power socket for the next cycle [5]. This process requires cognitive awareness as well as motor functions to be able to perform the charging process. The frequency and duration of recharging depend on the individual energy needed to provide effective therapy. Some novel stimulation regimens with higher frequencies up to several thousand Hertz may require more energy than traditional tonic stimulation. To date, there are no guidelines as to which patients with an indication for SCS therapy should be treated with a rechargeable device. To our knowledge, literature on r-IPG is limited, apart from studies that mainly focus on the cost-effectiveness of r-IPGs versus nr-IPGs [1, 4]. Therefore, the decision for or against a r-IPG is mostly influenced by the clinician’s perspective and experience. Moreover, the decision to change non-rechargeable versus r-IPG could be made by the previous stimulation regiment.

Moreover, the only patient-centric study which evaluated the therapy and convenience of r-IPGs (with only one IPG model) was published back in 2013 [6].

The present study uses a questionnaire-based approach to investigate the following aspects of r-IPGs from the patients’ perspective: convenience of recharging, user confidence and satisfaction, assessment of the burden of recharging and documentation of complications such as number of failed recharges and unintentional interruptions of therapy.

## Materials and methods

### Trial

The Recharge Pain Trial was conceived as a monocentric, questionnaire-based observational study.

### Patients

All adult (age ≥18 years) patients that had received SCS therapy for a certified indication to treat chronic pain syndromes with a r-IPG implanted at the time of the trial for at least 3 months were eligible for study participation. All patients were psychologically screened. Patients with untreated or uncontrolled psychiatric disorders were and are excluded for SCS therapy. The r-IPGs could be implanted during the initial SCS surgery (primary r-IPG) or during an IPG replacement surgery (secondary r-IPG).

### Design and procedure

A study kit including written trial information, the informed consent form, the questionnaire, and a stamped return envelope was sent to patients meeting the inclusion criteria. Patients who returned a questionnaire with a signed informed consent form within 6 weeks were included for data analysis. Data from the questionnaires was pseudonymized and transferred to a trial database in Microsoft ® Excel Version 16.55 (Microsoft Corporation, Redmond, WA, USA). Further clinical information (e.g., diagnosis, primary or secondary implant, localization of stimulator, date of surgery) was obtained from hospital charts.

### Questionnaire design

The questionnaire included 39 questions covering relevant aspects of handling a rechargeable SCS device (see Appendix). Questions were either open or closed with two or more possible answers. Convenience of charging, and overall effort were rated on an ordinal scale from 1 to 5 (1 = very hard/very high, 2 = hard/high, 3 = neutral, 4 = easy/low, 5 = very easy/very low). Regarding psychological influences of this type of therapy on patients, patients should rate their degree of agreement or disagreement on an ordinal scale from 1 to 5 (1 = “complete agreement,” 2 = “agreement,” 3 = “neutral,” 4= “disagreement,” 5= “complete disagreement”).

### Statistical analysis

Descriptive statistics (mean, standard deviation, range) were used for continuous variables (e.g., age). The median was calculated when testing on an ordinal scale (e.g., convenience of recharging). To test for significant differences between two means of continuous variables (e.g., mean age and gender) a Student’s *t*-test was performed. A univariate analysis of variance (one way ANOVA) was performed when investigating for difference between three or more independent continuous or discontinuous variables (e.g., mean age and type of stimulator). Continuous variables were tested for normal distribution using the Shapiro-Wilk test.

To test for significant differences between differences in frequency distributions of two or more discontinuous variables, the Pearson chi-square test was performed.

To test for significant differences in ratings of the five steps for convenience on an ordinal scale (dependent samples), a Friedman test was performed. When testing two different groups (e.g., primary or secondary stimulator) for significant difference in convenience, the Mann-Whitney *U* test was performed. For more than two groups, the Kruskal-Wallis analysis and post hoc Bonferroni comparison were performed. To test for significant linear correlations of two continuous variables (e.g., duration of charging and charging intervals) Pearson correlations were performed.

Questionnaires with incomplete data were still included in data analysis. Incomplete data (*n* < 40) and adjusted figures with existing data are marked with stern (*) in results. Statistical tests were then conducted in the same fashion but with a correspondingly adjusted total number of patients that answered the said questions. Based on a similar multicentric trial on r-IPGs for deep brain stimulation (DBS) therapy in movement disorder patients [5] (1), we unified the results of individual charging durations and intervals between recharges and calculated the “charge burden” (the amount of minutes (min) a patient has to invest per week to recharge the device):

$$\mathrm{Charge}\;\mathrm{Burden}\left(\frac\min{\mathrm{week}}\right)=\frac{\mathrm{duration}\;\mathrm{of}\;\mathrm{recharge}\;(\min)}{\mathrm{interval}\;\mathrm{between}\;\mathrm{recharges}\;\left(\mathrm{days}\right)}\ast7$$

Level of significance was defined as *p* < 0.05 in all cases. Confidence intervals were set at 95%. Statistical analysis was performed using IBM SPSS (IBM Corp. Released 2017. IBM SPSS Statistics for Windows, Version 25.0. Armonk, NY: IBM Corp.).

### Outcome parameters

Primary endpoint of this study was the convenience of the charging process. Secondary endpoints of this study were detection of complications with the r-IPG system, charge burden, the rates of user confidence and satisfaction, rate of complications (number of failed recharges and unintentional interruptions of therapy).

The impact of certain factors on the endpoints (e.g., pursuing a job, driving a car, use of electronic devices, opioid medication) was assessed.

Endpoints were furthermore analyzed for significant differences for several subgroups (age groups, gender, localization of pain and the position of the stimulator, type of the stimulator, timepoint of the implantation of the RC system (primary or secondary)).

### Ethical considerations

The investigation was conducted in accordance with the Declaration of Helsinki. The study protocol was reviewed and approved by the local Ethics Committee. The trial was registered in the German Clinical Trial Register (DRKS00021281).

## Results

### Study population

A study kit was sent to *n* = 96 patients meeting the inclusion criteria. A total of *n* = 40 patients (42%) returned the questionnaire with a signed informed consent form in time and were eligible for data analysis. The mean age at the time of the survey was 57.2 ± 12.6 years (range 33–82 years).

Patients *n* = 23 (57.5%) suffered from chronic pain after spinal surgery (CPSS) as the most common diagnosis in our study cohort. *n* = 7 patients (17.5%) suffered from complex regional pain syndrome (CRPS) type I and II. *n* = 5 (12.5%) suffered from chronic painful polyneuropathy. *n* = 2 (5%) patients suffered from other non-classified chronic pain syndromes. *n* = 1 (2.5%) suffered from angina pectoris (Table 1).

**Table 1:** Description of studied population

Variable	No. (%) of patients
Age (years)	
<40	5 (12.5%)
40–59	22 (55%)
60–79	13 (32.5)
Gender	
Male	23 (57.5%)
Female	17 (42.5%)
Diagnosis	
Chronic pain after spinal surgery (CPSS)	23 (57.5%)
Complex regional pain syndrome (CRPS) type I and II	7 (17.5%)
Chronic painful polyneuropathy	5 (12.5%)
Non-classified chronic pain syndrome	2 (5%)
Angina pectoris	1 (2.5%)
r-IPG model	
Medtronic Intellis™	13 (32.5%)
Medtronic Restore Sensor®	8 (20%)
Medtronic Restore Ultra®	18 (45%)
Timepoint of r-IPG implantation	
Primary	30 (75%)
Secondary	10 (25%)
Location of r-IPG	
Abdominal	22 (55%)
Gluteal	18 (45%)

Regarding psychiatric comorbidities in our cohort, which were accordingly moderate and controlled, 15% of patients reported being diagnosed with depression and 5% with an anxiety disorder.

The mean duration of SCS therapy with the r-IPG at the time of the survey was 52.1 ± 32.6 months (range 6–125 months). *n* = 10 patients (25%) with a secondary r-IPG had on average 90.4 ± 65.74 months (range 6–196 months) of SCS therapy before switching to the rechargeable system.

All patients included were implanted with Medtronic® r-IPG. 45% (*n* = 18) of patients were implanted with Restore Ultra® model, 32.5% (*n* = 13) of patients were implanted with Intellis^TM^ model and 20% (*n* = 8) of patients were implanted with Restore Sensor® model. Concerning the location of the r-IPGs, in 55% (*n* = 22) of the patients, the r-IPGs were implanted abdominal and 45% (*n* = 18) gluteal.

### Life with a rechargeable SCS device

All patients (*n* = 40) handled, monitored, and recharged the device themselves. 55% of patients (*n* = 22) received more than one training session in monitoring and operating the r-IPG after implantation. 89.7% (*n* = 35)* of patients felt sufficiently prepared after the final training session. The mean duration of stimulation was 21.2 ± 6.0 (SD) hours each day. The mean time interval of checking the battery capacity was 5.39 ± 4.0 days (range 1–18 days)*. On average, patients charged the stimulator every 10.24 ± 7.82 days (range 1–45)* and the charger every 17.24 ± 11.1 days (range 1–45)*. There was a significant positive correlation between the charging interval and the duration of recharging the stimulator (*p* = 0.02).

47.4% (*n* = 18)* patients charged the stimulator at a remaining battery level of 25–50%. 42.1% (*n* = 16)* patients charged the stimulator at a remaining battery level of less than 25%. *n* = 2 (5.3%)* patients each reported charging at a battery status of 50–75% or at a warning.

During the recharge process, 60.5% (*n* = 23)* patients remain stationary. Most patients (80.6%)* spent the time consuming media (watching TV or reading). 78.9% (*n* = 30)* patients had travelled since the implantation of the RC system.

### Recharging process

**Fig. 1:**
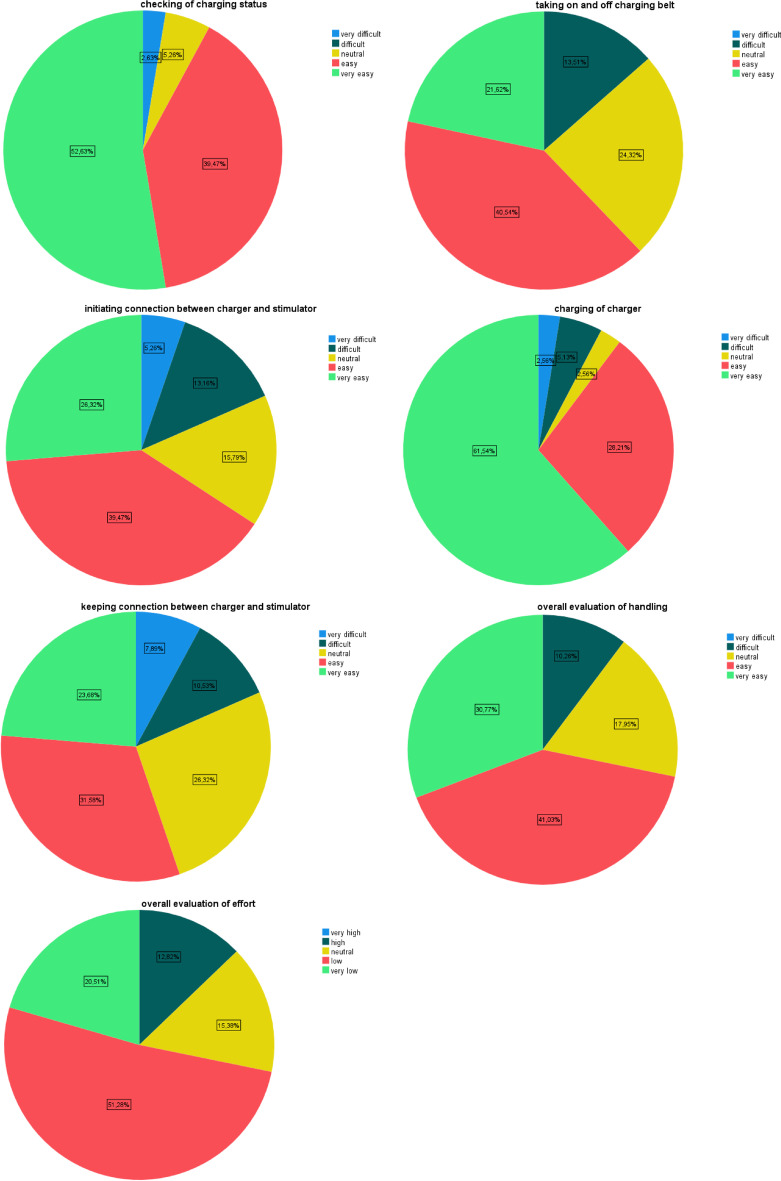
Rating of the recharging process

The overall convenience of recharging and burden of using the r-IPG were rated as “easy” and “low” with a median score of 4.0 points*. Individual steps of the recharging process were rated as “easy” (putting on charging belt, initiating connection, and keeping connection between charger and stimulator) with a median score of 4.0 points* or as “very easy” (checking charging status and charging of the charger) with a median score of 5.0 points* (Fig. 1).

“Checking the battery status” was rated more convenient than “putting on the charging belt” (*p* = 0.018), “initiating the connection” (*p* = 0.018) and “keeping the connection” (*p* = 0.002). “Charging the charger” was rated more convenient than “initiating the connection” (*p* = 0.018) and “keeping the connection” (*p* < 0.001).

Regarding the subgroup analysis, only the stimulator model did show significant influence on the overall convenience of recharging (*p* = 0.05) but not on the overall effort (*p* = 0.234). Patients with a Medtronic Intellis™ stimulator rated the convenience of recharging better than patients with the two other stimulator models (Medtronic Restore Sensor® and Restore Ultra®). The timepoint of the implantation of the RC system (primary or secondary r-IPG). User confidence did not have a significant influence on the overall rating (convenience of recharging). Working, driving and use of other rechargeable devices also did not significantly influence the overall evaluation of the recharging process. The use of opioid pain medication also did not have a significant impact on overall convenience of recharging.

According to patient expectations, non-rechargeable stimulators should last for a mean time of 8.9 years ± 4.5 (range 5–10.2 years) to be seen as a viable alternative or r-IPGs.

### Charge burden

The mean charge burden was 113 ± 140 min/week (range 24–840)* with a median of 63 min/week needed to recharge the device. There were no significant differences among the subgroups (age groups, gender, localization of pain, type of neurostimulator or position of stimulator) detected with regards to the charge burden (Table 2).

**Table 2:** Rating of overall handling, overall effort and charge burden

Subgroup	Overall convenience	Overall effort	Charge burden (min/week)	
	Median	Median	Mean ± SD	Median (range)
Total			112 ± 139	63 (42–135)
Age in years				
<40	4.0 (easy)	3.5 (neutral – easy)	46 ± 12	48 (30–60)
40–59	4.0 (easy)	4.0 (low)	139 ± 178	76 (28–640)
60–79	4.0 (easy)	4.0 (low)	90 ± 62	60 (24–210)
>80	3.0 (neutral)	3.0 (neutral)	70	
Gender				
Male	4.0 (easy)	4.0 (low)	80 ± 58	60 (24–210)
Female	4.0 (easy)	4.0 (low)	148 ± 190	105 (26–840)
Pain localization				
Legs	4.0 (easy)	4.0 (low)	84 ± 58	60 (24–210)
Back and legs	4.0 (easy)	4.0 (low)	182 ± 257	60 (42–840)
Other localization	4.0 (easy)	4.0 (low)	133 ± 70	120 (70–210)
Localisation of neurostimulator				
Abdominal	4.0 (easy)	4.0 (low)	115 ± 177	60 (24–840)
Gluteal	4.0 (easy)	4.0 (low)	105 ± 74	75 (26–240)
Type of neurostimulator				
Intellis	5.0 (very easy)^#^	4.0 (low)	81 ± 60	60 (24–210)
Restore Sensor	4.0 (easy)	4.0 (low)	81 ± 64	60 (28–210)
Restore Ultra	4.0 (easy)	4.0 (low)	157 ± 200	105 (30–840)
Working				
Yes	4.0 (easy)	4.0 (low)	109 ± 77	70 (42–240)
No	4.0 (easy)	4.0 (low)	111 ± 161	60 (28–840)
Driving				
Yes	4.0 (easy)	4.0 (low)	96 ± 63	68 (24–240)
No	4.0 (easy)	4.0 (low)	196 ± 359	38 (28–840)
Use of electronic rechargeable devices				
Yes	4.0 (easy)	4.0 (low)	86 ± 60	60 (24–240)
No	4.0 (easy)	4.0 (low)	373 ± 410	210 (70–840)

^#^ Significant difference within the subgroup (*p* < 0.05)

The associated factors (working, driving and use of rechargeable devices, opioid medication) did not have any significant impact on the charge burden either.

Patients taking opioid pain relievers had a significantly higher charge burden (*p* = 0.03) with a mean charge burden of 145.4* min/week per week compared to 61.3* min/week in patients not taking any opioids.

76.9% (*n* = 30)* of patients were satisfied with their charge burden.

### User confidence and user satisfaction

92.3% (*n* = 36)* of patients felt confident using the r-IPG at the time of the trial. The mean time until patients felt confident was 2.45 ± 1.33 weeks (range 1–4 weeks). Having only one training session did not have a significant impact on the confidence (*p* = 0.40). Non-confident users did report significantly higher rates of failed recharges (*p* = 0.05) but did not suffer from unintentional interruptions of therapy because of battery depletion (*p* = 0.20).

71.8% (*n* = 28)* felt that recharging time enabled them to actively participate in their therapy. 97.2% (*n* = 35)* would recommend a rechargeable system to another patient. 91.7% (*n* = 33)* would choose the rechargeable stimulator device again over a non-rechargeable one. Taking opioids did not have a significant impact on the recommendation (*p* = 0.38) or revised decision (*p* = 0.66) for the neurostimulator.

### Device-related satisfaction

While the implant volume of r-IPGs is smaller compared to non-rechargeable ones, the implant can still cause cosmetic issues. While most patients rated the size neutrally (47.5%), twice as many patients (35%) regarded the device to be small, rather than big (17.5%).

Recently, SCS systems with extracorporeal rechargeable and interchangeable pulse generators and wireless transfer of stimulation to the implanted lead have been introduced [7]. When being presented with both options to treat their condition, 82.5%* of patients preferred the r-IPG over an extracorporeal solution.

### Complications

37.5% (*n* = 15) of patients reported problems with recharging which resulted in an interruption of stimulation in 28.9% (*n* = 11)* of patients. In 43.2% (*n* = 16)* of cases, parts of the recharging system had to be replaced. Only 12.9% (*n* = 5)* patients reported fear of forgetting recharging.

No significant differences in the rate of complications were detected for any of the subgroups (age, gender, pain localization, position of neurostimulator, the timepoint of implantation and the use of opioids) and associating factors (pursuing a job, driving, use of rechargeable devices) except the model of r-IPG. Patients using the stimulator model Medtronic Intellis™ reported significant fewer interruptions of stimulation than patients using the stimulator model Medtronic Restore Sensor® or Restore Ultra® (*p* = 0.024). However, there were no significant differences in failed recharges between different IPG models (*p* = 0.210).

Patients who performed the recharge with a remaining battery capacity of less than 25% were not more likely to encounter problems with charging but had significantly more interruptions of therapy (*p* = 0.007).

*Incomplete data (*n* < 40) and adjusted numbers with existing data

## Discussion

The Recharge Pain Trial is the largest, industry-independent, and patient-centric study that included chronic and neuropathic pain patients treated with SCS system utilizing different r-IPG models. Our results demonstrate that the overall convenience of the recharging process was evaluated as “easy.” This result was also reflected in high rates of satisfaction and recommendation. The effort was not only rated as low, but 71.8% of the patients perceived it as active participation in their therapy.

However, failed recharge rate (37.5%) and interruption rate in stimulation therapy (28.9%) are very high compared to a study that examined r-IPGs in DBS [5]. The reason may be due to the difference in localization of the neurostimulator in DBS patients (predominantly subclavicular) versus SCS patients (predominantly abdominal or gluteal) which may be a better localization for recharging. Interruptions of the stimulation therapy were significantly lower with Medtronic’s newer generation stimulator, the Intellis™ model, than with Medtronic’s first-generation r-IPG models. In this regard, this proves that the technology has made meaningful improvements over time. Interruptions of therapy were significantly more common in patients who only recharged the r-IPG when the battery capacity was less than 25%. Therefore, patients should be instructed to regularly check their battery status and recharge at higher remaining capacities. “Keeping the connection” seems to be the most difficult step in the recharging process and “charging the charger” the easiest step. Similar results have been shown with r-IPG for DBS, which can be explained by the general similarity between DBS and SCS charging systems. Therefore, developing systems that make it easier and more reliable to keep the connection during recharging should be most urgently addressed by manufacturers [5].

The theory was put forward that handling smartphones or tablets that need regular battery checks recharging can be associated with higher convenience and satisfaction with r-IPGs for DBS [8]. However, this could not be found in a multicenter study with a longer follow-up in movement disorder patients [5]. Our study comes to the same conclusion. This may be due to different charging techniques (inductive versus wire charging) and different implications of charging a medical versus a non-medical device.

One common conception with r-IPGs is that elderly patients may have more trouble operating them. While in DBS therapy, there is reported to be a negative correlation with ratings of r-IPGs and age, this seemed to be non-existent anymore with a long-term follow-up [5]. In our study, we could not find a significant influence of age on the overall rating of convenience of the recharging process in the long-term monitoring. The use of new technologies such as rechargeable devices (e.g., smartphone or tablet) is not discouraged for older age groups. Finally, being of higher age should not be a principal contraindication for the application of r-IPGs for SCS therapy, which keeps the choice for different SCS devices very large, as some manufacturers now only offer rechargeable stimulators.

To our knowledge, we present for the first time the charge burden of r-IPG in SCS therapy. With less than 2 h per week needed to recharge and maintain therapy, we can better advise our patients, which is very relevant for the decision from the patients’ point of view. This charge burden is approximately the same as for movement disorder patients with rechargeable DBS systems — with different patient numbers and mid-term follow-up [5].

We found a significantly higher rate of failed recharges in patients that felt not confident using the device. User confidence showed no significant correlation with the number of training sessions received in handling the device though. Therefore, patients should actively be asked during follow-up visits and before they are discharged home after implantation whether they feel confident with the recharging process and receive another training session if necessary.

## Limitations

Decision to implant a r-IPG was based on patient preference and physician recommendation but not by utilizing any standardized approach. Since the MRI-compatible r-IPGs were mainly and firstly offered by Medtronic® in the early years of r-IPG development, patients at our hospital mainly received Medtronic® r-IPGs. For this reason, we only received the questionnaires from patients with Medtronic® r-IPG although patients with models from different manufacturers fulfilled the inclusion criteria and had received the study kit. Therefore, we could only compare the models of this company as a limitation. A comparison of r-IPGs from different manufacturers would of course be very interesting. A prospective and randomized study with equal and large sample sizes is required to find out whether user satisfaction and impact on quality of life really differ between rechargeable and non-rechargeable systems or between different r-IPG models. The stimulation parameters used could provide additional information on performance, efficiency, and capacity reduction of different r-IPG models and thus play an important role in the decision for or against r-IPG. This study did not include any patients that were converted from a r-IPG back to a non-rechargeable one. Identifying those patients that “fail” a r-IPG may offer important information on limitation of the said systems. The lack of return of questionnaires (only 40% of the questionnaires sent), which is not unusual for this type of study, and incomplete questionnaires result is a gap in the data, which negatively affects the validity of the data.

## Conclusion

Rechargeable IPGs for SCS therapy are generally rated as user-friendly and easy to manage from the patients’ perspective. However, the high rate of problems with recharging or interruption of stimulation, which is more pronounced in older IPG generations, shows the need for even further technical improvement and simpler handling. Some patient characteristics such as age or experience with a smartphone do not play a relevant role for or against an r-IPG.

### Supplementary Information

Below is the link to the electronic supplementary material.

Supplementary file1 (DOCX 34.0 KB)

## Data Availability

Data beyond the manuscript an appendix is not publicly available but can be requested from the corresponding author.
