# Supplementary material for: A single-center, open-label trial on convenience and complications of rechargeable implantable pulse generators for spinal cord stimulation: The Recharge Pain Trial
Source: Neurosurg Rev. 2023 Jan 14;46(1):36. doi: 10.1007/s10143-022-01940-y (PMC9840575; doi:10.1007/s10143-022-01940-y)
Supplement: Supplementary file 1 — Supplementary file1 (DOCX 34.0 KB) [file 10143_2022_1940_MOESM1_ESM.docx]

Appendix The additional Questionnaire with 39 questions and answers

| Question | Answers |  |
| --- | --- | --- |
| 1. Confident handling? * | Yes  No | *Absolute/relative number*  36 (90.0 %)  3 (7.5 %) |
| 1. Reason for implantation of r-IPG? * | Generator change  Pain  Others | *Absolute/relative number*  11 (27.5 %)  15 (37.5 %)  14 (35.0 %) |
| 1. Person making the decision for the r-IPG System? | Patient  Doctor  Family  Nurse | *Absolute/relative number*  13 (32.5 %)  27 (67.5 %)  0  0 |
| 1. Number of instructions given? | 1 instruction  >1 instructions | *Absolute/relative number*  18 (45 %)  22 (55 %) |
| 1. Sufficiently prepared after last instruction? * | Yes  No | *Absolute/relative number*  35 (87.5 %)  4 (10.0 %) |
| 1. Interval of recharging charger (days) |  | *17.24 ± 11.10 (mean ± SD)* |
| 1. Interval of checking battery status (days)? |  | *5.39 ± 4.00 (mean ± SD)* |
| 1. Interval of recharging stimulator (days) |  | *10.24 ± 7.82 (mean ± SD)* |
| 1. Charging at what battery level? * | <25 %  25-50 %  50-75%  >75 %  warning signal | *Absolute/relative number*  16 (40.0 %)  18 (45.0 %)  2 (5.0 %)  0 (0.0 %)  2 (5.0 %) |
| 1. Duration of charging per stimulator (minutes) |  | *107 ± 58 (mean ± SD)* |
| 1. Pleased with time required for charging? * | Yes  No | *Absolute/relative number*  30 (75.0 %)  9 (22.5 %) |
| 1. Acceptance of recharging time per week * | 15 – 30 minutes  30 – 60 minutes  60 – 120 minutes  120 – 180 minutes  > 180 minutes | *Absolute/relative number*  *3 (7.5 %)*  *7 (17.5 %)*  *21 (52.5 %)*  *7 (17.5 %)*  *1 (2.5 %)* |
| 1. Checking charging status of stimulator? | Patient  Family  Nurse  Other | *Absolute/relative number*  40 (100.0 %)  0  0  0 |
| 1. Person charging stimulator? | Patient  Family  Nurse  Other | *Absolute/relative number*  40 (100.0 %)  0  0  0 |
| 1. Stimulation time per day (hours) |  | *21.16 ± 6.00 (mean ± SD)* |
| 1. Mobility during charging? * | Yes  No | *Absolute/relative number*  15 (37.5 %)  23 (57.5 %) |
| 1. Activities during charging? * | Television  Reading  Working  Other activities | *Absolute/relative number*  24 (60.0 %)  5 (12.5 %)  3 (7.5 %)  2 (5.0 %) |
| 1. Travel after r-IPG implantation? * | Yes  No | *Absolute/relative number*  30 (75.0 %)  8 (20 %) |
| 1. Pursuing a job after r-IPG implantation? * | Yes  No | *Absolute/relative number*  11 (27.5 %)  27 (67.5 %) |
| 1. Driving after r-IPG implantation? * | Yes  No | *Absolute/relative number*  33 (82.5 %)  5 (12.5 %) |
| 1. Use of electronic devices? * | Yes  No | *Absolute/relative number*  35 (87.5 %)  3 (7.5 %) |
| 1. Use of opioid pain relivers? | Yes  No | *Absolute/relative number*  23 (57.5 %)  17 (42.5 %) |
| 1. Depression? | Yes  No | *Absolute/relative number*  6 (15.0 %)  34 (85.0 %) |
| 1. Anxiety disorder? | Yes  No | *Absolute/relative number*  2 (5.0 %)  38 (95.0 %) |
| 1. Problems with charging? | Yes  No | *Absolute/relative number*  15 (37.5 %)  25 (62.5 %) |
| 1. Interruption of stimulation? * | Yes  No | *Absolute/relative number*  11 (27.5 %)  27 (67.5 %) |
| 1. Evaluation of charging process? * | ***Checking of the charging state****:*  Very difficult  Difficult  Neutral  Easy  Very easy  **Taking on and off charging belt**  Very difficult  Difficult  Neutral  Easy  Very easy  ***Initiating connection between charger and stimulator:***  Very difficult  Difficult  Neutral  Easy  Very easy  ***Charging of charger:***  Very difficult  Difficult  Neutral  Easy  Very easy  ***Keeping connection between charger and stimulator:***  Very difficult  Difficult  Neutral  Easy  Very easy | *Absolute/relative number*  1 (2.5 %)  0 (0.0 %)  2 (5.0 %)  15 (37.5 %)  20 (50.0 %)  3 (7.5 %)  5 (12.5 %)  9 (22.5 %)  15 (37.5 %)  8 (20.0 %)  2 (5.0 %)  5 (12.5 %)  6 (15.0 %)  15 (37.5 %)  10 (25.0 %)  1 (2.5 %)  2 (5.0 %)  1 (2.5 %)  11 (27.5 %)  24 (60.0 %)  3 (7.5 %)  4 (10.0 %)  10 (25.0 %)  12 (30.0 %)  9 (22.5 %) |
| 1. Evaluation of the overall convenience of recharging? * | Very difficult  Difficult  Neutral  Easy  Very easy | *Absolute/relative number*  0 (0.0 %)  4 (10.0 %)  7 (17.5 %)  16 (40.0 %)  12 (30.0 %) |
| 1. Evaluation of the overall effort? * | Very high  High  Neutral  Low  Very low | *Absolute/relative number*  0 (0.0 %)  5 (12.5 %)  6 (15.0 %)  20 (50.0 %)  8 (20.0 %) |
| 1. Evaluation of the size of the stimulator? | Very big  Big  Neutral  Small  Very small | *Absolute/relative number*  0 (0.0 %)  7 (17.5 %)  19 (47.5 %)  14 (35.0 %)  0 (0.0 %) |
| 1. Charging the stimulator as a reminder of the disease? | Completely agree  Agree  Neutral  Disagree  Completely disagree | *Absolute/relative number*  5 (12.5 %)  3 (7.5 %)  12 (30.0 %)  11 (27.5 %)  9 (22.5 %) |
| 1. Charging the stimulator as active participation in therapy? * | Completely agree  Agree  Neutral  Disagree  Completely disagree | *Absolute/relative number*  12 (30.0 %)  16 (40.0 %)  10 (25.0 %)  1 (2.5 %)  0 (0.0 %) |
| 1. Fear of forgetting to charge? * | Completely agree  Agree  Neutral  Disagree  Completely disagree | *Absolute/relative number*  1 (2.5 %)  4 (10.0 %)  10 (25.0 %)  9 (22.5 %)  15 (37.5 %) |
| 1. Need to replace components of the Charge System after surgery? * | Yes  No | *Absolute/relative number*  *16 (40.0 %)*  *21 (52.5 %)* |
| 1. Recommendation of r-IPG? * | Yes  No | *Absolute/relative number*  *35 (83.3 %)*  *1 (16.7 %)* |
| 1. Preference of stimulator? * | Internal  External | *Absolute/relative number*  *33 (82.5 %)*  *2 (5.0 %)* |
| 1. Would choose r-IPG again? * | Yes  No | *Absolute/relative number*  *33 (82.5 %)*  *3 (7.5 %)* |
| 1. Importance of therapy? * | Fewer surgeries  Less charging | *Absolute/relative number*  *33 (82.5 %)*  *4 (10.0 %)* |
| 1. Expectation of non-rechargeable battery life |  | *8.90 ± 4.57 (mean ± SD)* |

*Incomplete data (n<40) and adjusted numbers with existing data
